# Advocacy for pneumonia prevention in Korea: a multi-dimensional program organised around World Pneumonia Day

**DOI:** 10.15172/pneu.2013.2/245

**Published:** 2013-04-10

**Authors:** Soon A. Kim, Paul E. Kilgore

**Affiliations:** 14 Translational Research Division, International Vaccine Institute, SNU Research Park, San 4-8 Nakseongdae-Dong, Kwanak Gu, Seoul, Korea 151-919; 24 Department of Pharmacy Practice, Eugene Applebaum College of Pharmacy & Health Sciences, Wayne State University, Michigan, USA

**Keywords:** pneumonia, advocacy, World Pneumonia Day, public-private partnership

## Abstract

There are limited examples of population-based approaches that engage a broad range of stakeholders for prevention of pneumonia. In 2010, a multi-dimensional public-private partnership was established around World Pneumonia Day (WPD) in Seoul, Korea and included the following components: a) formation of an expert advisory group, b) creation of educational materials tailored for lay persons, c) creation of a dedicated WPD internet website in the local language, d) organisation of a WPD venue in central Seoul, e) creation of video and social networking messages for wide distribution, and f) engagement of parents, health-care professionals, public health agencies and policymakers. This project directly engaged 7 expert health professionals, 5 national- and city-level health facilities, and parents from communities. The program reached out to 70,560 persons including 25,200 persons who were contacted in person at publicly-held WPD events. An educational video produced for WPD was aired in the Seoul subway and visible to several million persons riding subway lines that aired the pneumonia public service announcements over a two-month period (February to March, 2011). In addition, the Korean WPD website experienced 4,975 page views with 3,338 visitors and the micro blog associated with this site hosted 82 posts from site visitors. Based on participant numbers and contact volumes achieved in this project, the Korean WPD program was widely accepted and proved to be a highly effective in reaching a large audience to advocate for pneumonia prevention. One key to success of this program appears to be the unique public-private partnership around a major health issue. The methods and tools developed in this program have excellent potential for adaptation and application in other countries where pneumonia may be an under recognised problem among the general public.

## 1. Introduction

Globally, pneumonia is a devastating cause of morbidity and mortality, especially in children and the elderly [1, 2]. In 2005, the World Health Organization (WHO) estimated that 1.6 million people die of pneumococcal disease every year, including 700,000 to one million children aged 1–59 months [3]. To combat this immense burden of disease, pneumococcal conjugate vaccines (PCVs) have been developed and introduced [1, 4, 5]. In addition, PCVs are now available for use in older adults [6–9]. In Korea, several studies of pneumonia and pneumococcal disease show that both children and adults have substantial burden due to *Streptococcus pneumoniae*, at present, the use of PCVs in infants has not been paid by the government program [10, 11].

In conjunction with global efforts for pneumococcal vaccine development and introduction, there has been growing recognition among public health experts that community leaders, medical professionals, parents, and families may benefit from targeted health information to better understand the value of vaccines that prevent severe disease and death associated with pneumonia [12–14]. Since 2009, through the efforts of the Global Coalition against Child Pneumonia, World Pneumonia Day (WPD) has taken place on November 12 to encourage efforts among healthcare professionals, policymakers, the general public and donors to highlight the impact of pneumonia among local populations and to highlight challenges that remain in the fight to improve care for and prevent pneumonia [15–17].

To date, however, there have been limited efforts to integrate education, outreach and advocacy activities during WPD events that focus on increasing recognition of pneumonia with the long-term goal of improving the prevention of pneumonia [17–19]. To address this gap, we established an innovative public-private partnership in 2010 bringing together a wide range of stakeholders for pneumonia outreach, education and advocacy in South Korea in order to raise public awareness of the disease and to promote its prevention. Our experience provides lessons for the development of an integrated pneumonia advocacy tool kit that can be used in other populations.

## 2. Methods

### 2.1. Target population for advocacy, pneumonia education and outreach materials

**Figure 1.**
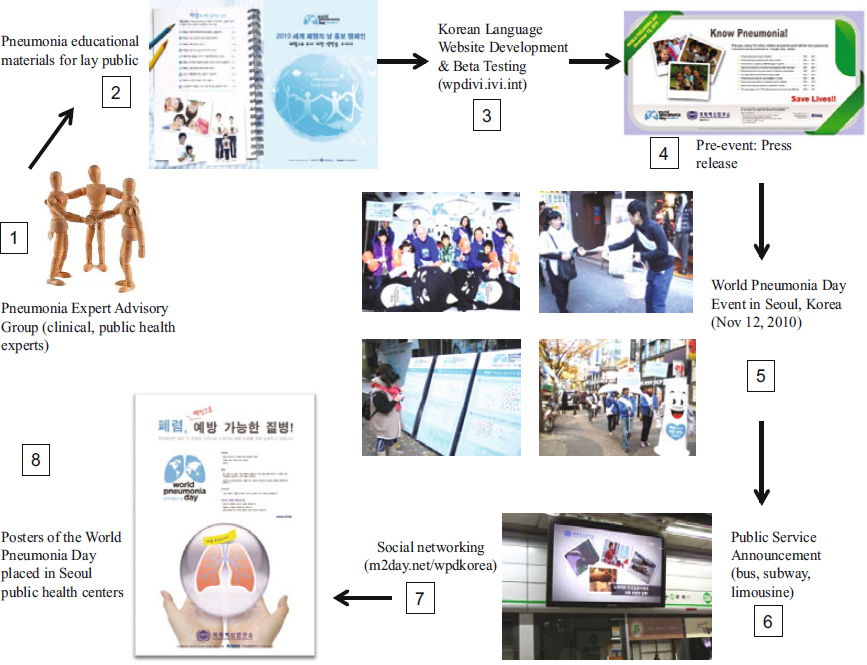
Major activities comprising the pneumonia advocacy program in Seoul, Korea.

Seoul is the largest metropolitan area in Korea and one of the largest in the world with a total population estimated at 10.4 million in 2010. Due to its large population, the presence of key stakeholder and policymaking organisations in Seoul and the broad influence that Seoul exerts on national policies, a downtown Seoul location was identified for the pneumonia advocacy program. The process of organising the pneumonia advocacy project in Seoul also enabled direct engagement of the Seoul city health department and the Office of the Mayor for the city of Seoul. Such support from the Office of the Mayor proved to be crucial to the success of the project and enhanced media attention on the WPD events held in Seoul. To establish collaborations, we first established an Expert Advisory Group (EAG) to serve as reviewers of educational content that was created for pneumonia outreach and educational programs. This review helped to ensure that all materials were consistent with current international and Korean clinical practice and prevention guidelines for pneumonia (Figure 1). Next, a convenience sample of laypersons was assembled to review pneumonia educational materials. These reviewers provided feedback on the format, layout, content and readability of the educational materials. The EAG also served as advisors for the production of the pneumonia public service announcements (PSAs) to ensure these messages contained state-of-the-art medical information.

In addition to ensuring involvement among government public health leaders in Seoul, the project team directly met with staff of the Guro District Health Center (located in southwest Seoul) and the health department of the Seoul Metropolitan Government as well as other district health centers located around Seoul. Inclusion of the public sector in the WPD activities broadened the overall outreach of this project to several hundred public health center staff in Seoul and enabled experienced public health professionals to provide expert input into the design and presentation of pneumonia messages in Korean language posters and pamphlets.

### 2.2. The World Pneumonia Day event

The WPD event was held in the Myeongdong area of Seoul on November 12, 2010. Myeongdong is one of the busiest shopping areas in Seoul with heavy foot traffic on a daily basis. The project team engaged an experienced event-organising firm and additional partnerships were established with WPD staff from the International Vaccine Access Center of the Bloomberg School of Public Health at Johns Hopkins University (Baltimore, USA) and the Global Alliance for Vaccines and Immunisations (GAVI)-Korea Partnership Project (Seoul, Korea).

To introduce WPD to the Korean public, the project team reached out to media outlets to place advertisements about WPD events in English and Korean language newspapers (e.g., The Korea Times). In addition, popular Korean Internet portals (e.g., www.naver.com) were engaged to display banner advertisements for the WPD events and an educational campaign with messages around the prevention of pneumonia. With expert input from the International Vaccine Institute’s (IVI; Seoul, Republic of Korea) information technology team, a new Korean language WPD website (http://www.wpdk.ivi.int/), modelled after the existing English WPD website, was developed to further promote WPD and provide a centralised repository of publicly-accessible pneumonia education and outreach materials.

To maximise audience exposure and attention on November 12, 2010, an event public information booth for the distribution of educational pamphlets and audience game participation was set-up directly adjacent to the event soundstage. Representatives from the local government, program partners, and local celebrities were present at the event to further increase attention from the media and public. The event organisers designed and implemented an entertaining visual demonstration that depicted children “fighting” against the germs of pneumonia and was performed onstage for media outlets and the public. After this stage performance, interactive outreach and educational activities were conducted throughout the day to engage and inform the public. At the event booth, passers-by were invited to play educational quiz games that involved placing stickers onto a board in response to ten questions about pneumonia (Table 1).

**Table 1:** Answers to questions about pneumonia from participants at the World Pneumonia Day event in Seoul, Korea on November 12, 2010.

Questionnaire Item	Answer	Correctly answered	Incorrectly answered
Pneumonia occurs only in children.	No	153 (92.7%)	12 (7.3%)
Pneumonia can spread only by direct contact.	No	143 (87.7%)	20 (12.3%)
Pneumonia is caused by different types of germs.	Yes	142 (78.5%)	39 (21.5%)
Signs and symptoms of pneumonia disappear after few days.	No	150 (88.2%)	20 (11.8%)
Pneumonia occurs only as a result of influenza complication.	No	215 (93.1%)	16 (6.9%)
It is very rare for pneumonia to cause death.	No	208 (92.9%)	16 (7.1%)
Pneumococcal vaccines are available in Korea.	Yes	154 (93.9%)	10 (6.1%)
Pneumonia vaccination is available only for children.	No	154 (96.3%)	6 (3.8%)
Adult pneumococcal vaccine is available in Korea.	Yes	129 (78.2%)	36 (21.8%)
The vaccinations for H1N1 influenza and pneumonia are different.	Yes	138 (86.3%)	22 (13.8%)

In addition, this event provided an opportunity for the general public to express comments and voice their support about pneumonia through micro-blogging using the popular Korean online social networking site, www.me2day.net. IVI staff, lay volunteers and a GAVI Korea representative distributed educational flyers to the public on the day of the event in downtown Seoul. Finally, volunteer youth messengers from local universities conducted outreach throughout the day by walking through the Myeongdong area holding WPD placards and passing out educational pamphlets.

### 2.3. Other advocacy programs

PSAs were produced with public health experts from the EAG who verbalised messages on the recognition, diagnosis, treatment and prevention of pneumonia, and they aired for passengers aboard 296 buses moving through 14 different routes between locations to drop-off and pick-up passengers at the major cities of Seoul Busan, Daegu and Incheon and also Gimpo and Incheon International Airports in cooperation with the private bus operating companies from February 16 through March 15, 2011. Finally, we distributed approximately 100 posters through the district community health centers to present the message of prevention for pneumonia.

### 2.4. Ethical approval

The study was exempt from human subjects ethics review because no experimental trial was undertaken and no personal/confidential information was collected.

## 3. Results

### 3.1. Multi-dimensional nature of the advocacy

We hypothesised that achieving a maximum population impact for the pneumonia advocacy activities would require active outreach to parents, professionals, government officials and other key stakeholders through every available means. Public partners included the Department of Health and Welfare of Seoul Metropolitan City, Seoul National University Hospital, a government district community health center, social welfare centers, and the Korean Medical Association. Logistically, based on priorexperience working on pneumonia projects in Korea and elsewhere, the project team was able to call upon key experts and local firms to execute discrete activities, including, creating and distribution of educational pamphlets, video production and distribution, website development, social networking site outreach and parent outreach.

### 3.2. Population impact of advocacy activities

Based on calculated foot traffic on November 12, we estimated that 25,200 individuals visited the WPD booth during a five-hour period and an additional 45,360 individuals received visual and auditory exposure to the WPD campaign slogans on the street adjacent to the WPD event venue, based on floating population per minute, then a total number of approximately 70,560 people were exposed to WPD. Data collection during the event was conducted by engaging people on the street to participate in an interactive game consisting of ten questions about pneumonia. Of the estimated 25,200 persons who visited the WPD venue, a total of 1,783 persons responded to questions concerning their perceptions and knowledge about pneumonia to which they responded by applying “yes” or “no” stickers on a question board. Among the ten questions asked, the ones that most elicited incorrect answers were: “Adult pneumococcal vaccine is available in Korea” (21.8%) and “Pneumonia is caused by different types of germs” (21.5%) (Table 1). These were followed by: “The vaccinations for H1N1 influenza and pneumonia are different” (13.8%), “Pneumonia can be spread only by direct contact” (12.3%), and “The signs and symptoms of pneumonia disappear after a few days” (11.8%).

The educational pneumonia PSA was produced, distributed and broadcasted on video screens in waiting areas and inside subway cars of two heavily-trafficked subway lines in Seoul. The same PSA aired on TV monitors within buses moving to and from Incheon International Airport. According to the Korean National Statistics Office, the number of daily average subway riders in Seoul is estimated to be nearly 5 million [20] and was used as a measure of the estimated exposures. The PSA also aired in the public subway systems of Busan, Daegu and Incheon.

## 4. Discussion

This project represents the first coordinated, multi-disciplinary pneumonia advocacy program conducted around WPD and this program also marked the first coordinated program to recognise WPD in Korea. The WPD event was successfully conducted in a bustling area of Seoul to raise public awareness of pneumonia, especially among mothers, teenagers, and young adults who frequent this area. According to a report from WPD 2010, more than 42 countries and thousands of advocates in Asia, Africa, Latin America, Europe and the United States came together on WPD to promote pneumonia awareness and save lives worldwide [21]. As a result, WPD empowered advocates across the globe to implement a wide range of creative strategies to inform and motivate policymakers and the public on the need to take action against pneumonia. In Korea, the campaign was well-received across both public and private sectors and no evidence was found to suggest the presence of negative sentiments or views toward the WPD campaign. The positive perceptions of the WPD and pneumonia advocacy program may, in part, reflect the extensive efforts that the project staff employed to engage a broad range of stakeholders as well as provide information that could be understood by lay persons in conjunction with well-recognised medical authorities and institutions. In the course of this project, a number of key lessons emerged. First, successful completion of the project required the involvement of individuals with a broad range of skill sets. The multi-disciplinary team assembled in this project included experts in clinical medicine, event planning, public health, educational, social-behavioral, communications, information technology, web design and video production. Second, the perception of key stakeholders that there is great value in a large, coordinated advocacy program for pneumonia required dedication, persistence, enthusiasm, fortitude and reassurance of project partners. A key factor in the success of this project was the extensive time dedicated to engaging prospective public and private partners, which proved to be crucial because an advocacy program for pneumonia has never been conducted before in Korea. Third, project coordination and communication among the diverse program partners requires careful management to ensure on-time completion of multiple and overlapping activities. Fourth, broad engagement of stakeholders across public and private sectors helped to identify additional partners who could join future WPD events and pneumonia advocacy activities.

In developing this project, the organisers engaged sponsors who sought to support a community-based project that would benefit the broader public in Korea. At the same time, this sponsor had no commercial interests related to products that specifically prevented, diagnosed or treated pneumonia in any age group. The lack of conflict of interest added additional credibility to the overall WPD campaign in Korea. In addition, the scope of this WPD program required substantial fund-raising and financial support. The overall cost of the program (∼$US 125,000) was perhaps higher to conduct in Korea due to Korea’s overall higher costs of living and doing business. Major costs in this program were incurred in the conduct of the daylong public WPD event in Myeong-dong, deployment of the Korean language WPD website and the production and distribution of the pneumonia PSA. It is important to note that a large number of collaborators participated in WPD activities without receiving compensation, including several district health officers and members of the Seoul Mayor’s office. Only members of the EAG received an honorarium for their participation and provision of technical advice.

In lower income countries, the conduct of events to recognise WPD should also be feasible and require less financial resources than the campaign in Korea. A multi-year WPD campaign may have higher costs at the start of a program with lower costs achieved through the reuse of tools, educational materials, internet sites and PSA materials each year. Collaboration with universities, hospitals and government agencies is also likely to reduce program costs as these organisations may dedicate personnel time provided voluntarily or at reduced cost. With appropriate planning and financing, replication of this program is highly feasible in other countries.

In conclusion, lessons from this novel program suggest that evidenced-based advocacy programs for the prevention of pneumonia and other diseases are feasible and acceptable in Korea. With the first WPD observance and pneumonia advocacy program completed in Seoul, the tools developed serve as excellent resources for future WPD programs to be conducted elsewhere.
